# Living myocardial slices as a model for testing cardiac pro-reparative gene therapies

**DOI:** 10.1016/j.ymthe.2025.03.033

**Published:** 2025-03-25

**Authors:** Rocco Caliandro, Azra Husetić, Merel L. Ligtermoet, Arie R. Boender, Lorena Zentilin, Gerard J.J. Boink, Mauro Giacca, Monika M. Gladka

**Affiliations:** 1 Department of Medical Biology, Amsterdam Cardiovascular Sciences, Amsterdam University Medical Centers, University of Amsterdam, Amsterdam, the Netherlands; 2 Department of Cell and Chemical Biology, Leiden University Medical Center, Leiden, the Netherlands; 3 PacingCure BV, Roetersstraat 35, 1018 WB Amsterdam, the Netherlands; 4 AAV Vector Unit, International Centre for Genetic Engineering and Biotechnology (ICGEB), Trieste, Italy; 5 Department of Cardiology, Amsterdam Cardiovascular Sciences, Amsterdam University Medical Centers, University of Amsterdam, Meibergdreef 9, 1105 AZ Amsterdam, the Netherlands; 6 School of Cardiovascular and Metabolic Medicine & Sciences, King’s College London, London, UK

**Keywords:** living myocardial slices, ZEB2, TMSB4, PTMA, endothelial cells, gene therapies

## Abstract

Available models currently adopted for preclinical studies in the cardiovascular field either fail to recapitulate human cardiac physiology or are extremely expensive and time-consuming. Translational research would greatly benefit from the development of novel models that reflect the native mature phenotype of the human heart while being cost and time effective. Living myocardial slices (LMSs) have emerged as a novel, powerful *ex vivo* tool for translational research. Although the number of studies adopting LMSs is rapidly increasing, this model remains largely under-characterized. In this study, we make use of LMSs and compare them to a murine model to deliver the cardioprotective factor zinc finger E box-binding homeobox 2 (ZEB2), a transcription factor known to exert cardioprotective effects after ischemic injury and promote the secretion of pro-angiogenetic factors thymosin beta-4 (TMSB4) and prothymosin alpha (PTMA). Our data show that viral-mediated delivery of these factors induced similar cardiomyocyte gene expression changes in LMS and mouse models. We also show that the delivery of these pro-angiogenic factors enhances an angiogenic response in both models, indicating that LMSs are a suitable alternative to mice for studying the effects of gene transfer in various cardiac cell types.

## Graphical abstract

**Figure:**
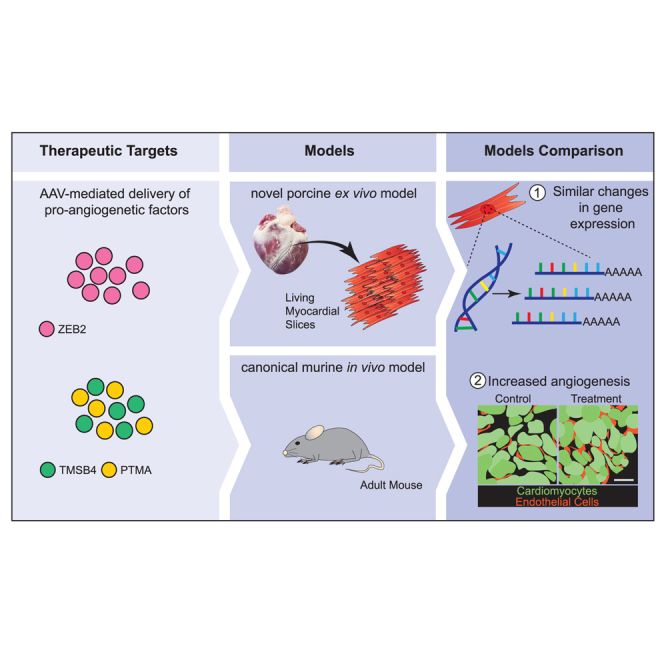

## Teaser

Living myocardial slices (LMSs) are a functional, cost-effective *ex vivo* model for testing cardiac gene therapies. This study shows that AAV6-mediated delivery of pro-regenerative factors ZEB2, TMSB4, and PTMA induces pro-angiogenic responses in LMSs, mirroring *in vivo* findings and advancing LMSs as a powerful tool for translational research.

## Introduction

Gene therapies for cardiac repair are typically tested in preclinical animal models due to the complexity of the healing process, which involves multiple pathways across different cell types. Rodent models fail to fully replicate human cardiac physiology,^1^^,^^2^^,^^3^ while pig studies, despite their similarities to humans,^4^ are costly and time-consuming. Living myocardial slices (LMSs) offer a cost-effective alternative, retaining tissue architecture and cell interactions, though they remain under-characterized.

Zinc finger E box-binding homeobox 2 (ZEB2) is a transcription factor that promotes cardiac repair. Our previous research showed that adeno-associated virus 9 (AAV9)-mediated delivery of ZEB2 to mice enhances angiogenesis, reduces scarring, and preserves heart function by stimulating the release of pro-angiogenic factors thymosin beta-4 (TMSB4) and prothymosin alpha (PTMA). Thus, delivering ZEB2, TMSB4, and PTMA is a promising strategy to promote cardiac repair.^5^^,^^6^

This study is the first to compare viral-mediated delivery of ZEB2, TMSB4, and PTMA in both LMSs and mice. A direct comparison of the transcriptomic data revealed similar gene expression changes in both models. Additionally, the delivery of pro-angiogenic factors to LMSs increased endothelial cell (EC) proliferation, mirroring the results in mice, thus confirming LMSs as a viable alternative for testing cardiac regenerative gene therapies.

## Results

### High-quality LMSs represent a valuable *ex vivo* platform for gene therapy testing

**Figure 1:**
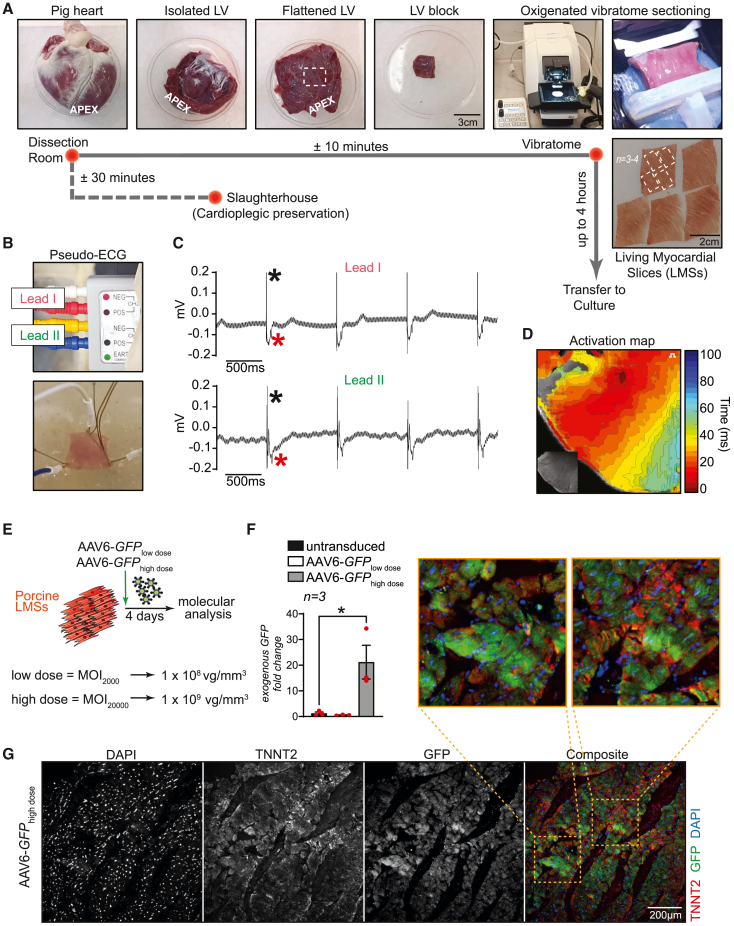
High-quality LMSs represent a valuable *ex vivo* platform for gene therapy testing (A) Experimental flow of LMSs preparation. The pig heart was collected from the slaughterhouse and underwent cardioplegic arrest, LV isolation, and 300-μm-thick sectioning using a vibratome (in line with the orientation of the fibers) and then was prepared for culturing. (B) A bipolar-ECG setup to follow LMSs responsiveness using a point electrode and stimulation of 1 Hz. (C) Representative pseudo-echocardiograms (4 s are shown) indicate electrical activation of LMSs (red asterisk) after electrical stimulation (black asterisk). (D) A representative isochrone activation map shows the propagation of an action potential along the LMSs fiber, following the point stimulation at 1 Hz (top right corner, white mark). (E) Experimental flow of AAV6-mediated gene transfer. LMSs were infected with AAV6-*GFP* at a low and a high dose. (F) The delivery of GFP was confirmed at the mRNA level with qPCR. (G) Immunofluorescence showing successful delivery of GFP in PFA-fixed LMSs treated with high-dose AAV6-*GFP*. Data are represented as mean ± SEM. Each dot represents a biological replicate, *n* = 3. ∗*p* < 0.05 using one-way ANOVA. Scale bar = 200 μm.

We generated LMSs from the left ventricle (LV) of slaughterhouse pigs after collecting and preserving the hearts post-mortem with hypothermic heparinized cardioplegic solution (Figure 1A). We produced 300-μm-thick, high-quality sections and tested them for viability. We performed pseudo-electrocardiography (ECG) with a point electrode and a bipolar ECG setup (Figure 1B) and monitored the tissue response (red asterisk) after 1 Hz electrical stimulation (black asterisk) (Figure 1C). Additionally, we assessed action potential activation using optical mapping. We incubated LMSs in Di-4-ANEPPS dye, paced them at 1 Hz, and used fluorescent live imaging to generate isochromatic activation maps, confirming that LMSs can propagate action potentials (Figure 1D). Next, we tested the gene-transfer capacity of LMSs using low- and high-dose AAV6-*GFP,* collecting samples for molecular analysis 4 days post-infection (Figure 1E). Based on previous studies,^7^^,^^8^ we estimated that ∼40,000–50,000 cardiomyocytes (CMs) per mm^3^ requires 1 × 10^8^ vg/mm^3^ for an MOI of 2,000 (low dose) and 1 × 10^9^ vg/mm^3^ for an MOI of 20,000 (high dose) of AAV (Figure 1E). Figure 1F shows no GFP delivery with low dose AAV6*-GFP* in LMSs, but a 10-fold higher dose led to robust GFP delivery, with a 20-fold mRNA increase compared to untreated samples. Immunofluorescence confirmed GFP overexpression in paraformaldehyde (PFA)-fixed LMSs, showing a mosaic-like pattern in CMs (Figure 1G), demonstrating that high-quality LMSs are functional and suitable for AAV-mediated gene transfer.

### AAV-mediated ZEB2 delivery shows similar transcriptional effects in LMS and mouse hearts

**Figure 2:**
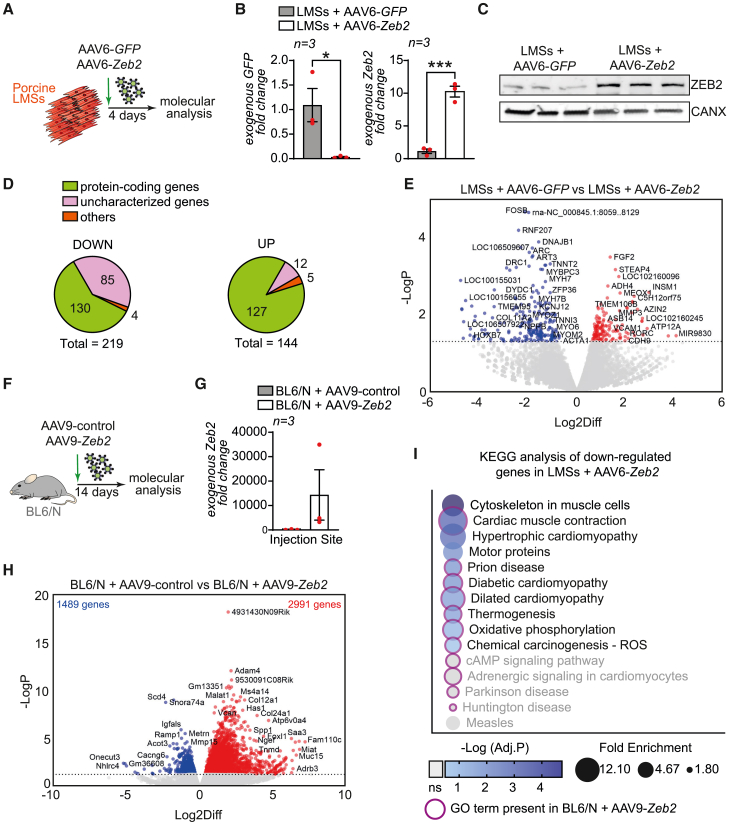
AAV-mediated ZEB2 delivery shows similar transcriptional effects in LMSs and mouse hearts (A) Schematic representation of the experimental setup in LMSs. (B and C) Confirmation of the delivery of *GFP* and *Zeb2* with (B) qPCR and (C) western blotting. (D) Pie charts showing numbers of down- and up-regulated genes identified by RNA sequencing (RNA-seq) and their classification. (E) Volcano plot showing up-regulated genes (red dots) and down-regulated genes (blue dots) in LMSs. Dotted line indicates −log*p* = 0.05. (F) Schematic representation of the experimental setup in mice. (G) Successful delivery of *Zeb2* was confirmed with qPCR. (H) Volcano plot showing up-regulated genes (red dots) and down-regulated genes (blue dots) in mice. Dotted line indicates −log*p* = 0.05. (I) KEGG pathway analysis of the down-regulated genes in AAV6-*Zeb2*-treated LMSs. Common pathways (from down-regulated genes) found in LMSs and mice treated with ZEB2 are indicated with purple circles. Data are represented as mean ± SEM. Each dot represents a biological replicate, *n* = 3. ∗*p* < 0.05, ∗∗∗*p* < 0.001 using Student's t test.

To assess porcine LMSs as an alternative to animal studies, we delivered *ZEB2* to LMSs and collected them for analysis 4 days post-infection (Figure 2A). ZEB2 delivery was confirmed at the RNA (Figure 2B) and protein (Figure 2C) levels. We performed RNA sequencing of LMSs treated with AAV6*-GFP* or AAV6-*Zeb2*, identifying over 360 significantly differentially expressed genes (Figures 2D and 2E). Most were protein coding, but nearly 30% were uncharacterized in the *Sus Scrofa 11.1* genome and excluded from downstream analysis due to annotation uncertainty (Figure 2D). To compare LMSs results with an *in vivo* mouse model, we delivered ZEB2 via intracardiac injection into the LV of adult BL6/N mice (Figure 2F). Fourteen days post-injection, we confirmed the delivery of *Zeb2* with quantitative PCR (qPCR) (Figure 2G) and performed RNA sequencing of LV. We identified nearly 4,500 differentially expressed genes (Figure 2H). KEGG analysis of down-regulated genes found in mice were linked to cardiac muscle contraction, cytoskeleton organization, and cardiomyopathies, similar to those found in LMSs (Figure 2I, purple circles), indicating LMSs as a viable alternative to mouse studies.

### Pro-angiogenic gene delivery enhances EC proliferation in LMSs

**Figure 3:**
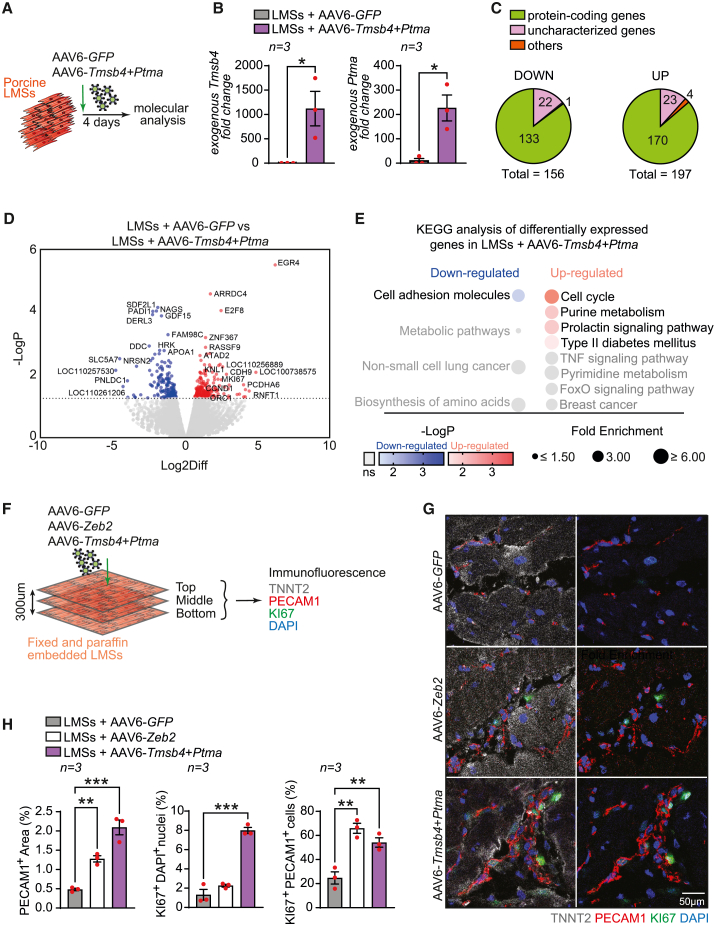
Pro-angiogenic gene delivery enhances EC proliferation in LMSs (A) Schematic representation of the experimental setup in LMSs. (B) Confirmation of the delivery of *Tmsb4* and *Ptma* with qPCR. (C) Pie charts showing numbers of down- and up-regulated genes identified by RNA-seq of AAV6-*Tmsb4+Ptma*-treated LMSs and their classification. (D) Volcano plot showing up-regulated genes (red dots) and down-regulated genes (blue dots) in AAV6-*Tmsb4+Ptma*-treated LMSs. Dotted line indicates −log*p* = 0.05. (E) KEGG pathway analysis of differentially expressed genes in AAV6-*Tmsb4*+*Ptma-*treated LMSs showed up-regulation of cell-cycle-related genes and down-regulation of cell adhesion molecules. (F) Assessment of angiogenic response after treatment with AAV6-*GFP*, AAV6-*Zeb2*, or AAV6-*Tmsb4+Ptma* for 4 days. (G) Immunofluorescence analysis of PFA-fixed LMSs highlighted an increase in PECAM1+ area and KI67+ nuclei. (H) Quantification of PECAM1+ areas, KI67+ nuclei, and KI67+ PECAM1+ cells in AAV6-*GFP*-, AAV6-*Zeb2*-, or AAV6-*Tmsb4+Ptma*-treated LMSs. Data are represented as mean ± SEM. Each dot represents biological replicate, *n* = 3. ∗*p* < 0.05, ∗∗*p* < 0.01, ∗∗∗*p* < 0.001 using Student's *t* test or one-way ANOVA. Scale bar = 50 μm.

Our previous study shows that ZEB2 delivery to CMs regulates the secretion of pro-angiogenic proteins TMSB4 and PTMA, promoting EC proliferation and migration.^6^ Therefore, we added an LMSs group treated with a combination AAV6-*Tmsb4* and AAV6-*Ptma* (AAV6-*Tmsb4*+*Ptma*). ZEB2 or TMSB4+PTMA were delivered to fresh LMSs, and samples were collected 4 days later for analysis (Figure 3A). TMSB4 and PTMA delivery was confirmed by qPCR (Figure 3B), and RNA sequencing was performed on LMSs treated with AAV6-*GFP* or AAV6-*Tmsb4*+*Ptma*. We identified over 300 significant differentially expressed genes in AAV6-*Tmsb4*+*Ptma*-treated LMSs, with most genes being well annotated in the *Sus Scrofa 11.1* genome (Figures 3C and 3D). KEGG pathway analysis of differentially expressed genes in LMSs revealed “cell cycle” as the top up-regulated term and “cell adhesion molecules” as the top down-regulated term, suggesting activation of proliferation and migration (Figure 3E). Next, we performed immunofluorescence on PFA-fixed LMSs to detect the EC marker PECAM1 and the proliferation marker KI67 (Figure 3F). Both AAV6-*Zeb2* and AAV6-*Tmsb4+Ptma* treatments increased PECAM1-positive areas, indicating induced angiogenesis (Figures 3G and 3H). While ZEB2 showed a minor rise in proliferating nuclei in general, it increased KI67-positive ECs. Yet, AAV6-*Tmsb4+Ptma* delivery led to an 8-fold increase in KI67-positive nuclei, mostly in PECAM1-positive cells. These results show that the delivery of ZEB2, TMSB4, and PTMA induces EC proliferation, making LMSs a valuable model for studying the angiogenic potential of gene therapies.

## Discussion

Our study highlights the potential of LMSs as a high-quality, functional *ex vivo* platform for testing gene therapies, offering a significant step forward in bridging the gap between traditional *in vitro* and *in vivo* models. By generating LMSs from porcine hearts, we demonstrated tissue viability and functionality through pseudo-ECG recordings and optical mapping. We validated the feasibility of AAV-mediated gene transfer in LMSs, yielding a mosaic-like transduction pattern in CMs, which was sufficient to assess the downstream pro-angiogenic effects of ZEB2 and TMSB4+PTMA delivery. While more uniform transduction efficiency is often desired, the question remains whether this is needed to yield a therapeutic effect. Studies using AAV-based delivery suggest that even modest transduction efficiency (40%–65%) can prevent arrhythmogenesis in inherited arrhythmia syndromes.^9^^,^^10^ Qi et al. recently demonstrated that correcting 20% of cardiomyocyte transcripts via AAV-based base editing fully prevented ventricular tachycardia in an LQT3 mouse model.^11^ This suggests that even limited cardiomyocyte transduction may be enough to partially slow disease progression. Nevertheless, researchers continue to refine AAV vectors to enhance efficiency in applications demanding high transduction levels.

A key advantage of the LMSs model is its potential to reduce animal use in preclinical research by serving as an intermediate platform for early-stage screening of drug candidates and gene therapies. LMSs can be a valuable platform for optimizing AAV vectors and assessing gene-transfer capacity, cell specificity, and dose-dependent effects of viral delivery systems. The broad applicability of LMSs as an intermediate testing platform aligns with the 3R principles (reduce, reuse, recycle) by minimizing early animal use, optimizing candidate selection before *in vivo* studies, and reserving animal experiments for validating the most promising therapeutic targets.

LMSs technology has certain limitations, including relatively short-term viability in the traditional air-liquid interface. Extending culture duration requires specialized platforms that provide continuous mechanical load and electrical stimulation. Furthermore, our transcriptomic analysis revealed an overrepresentation of cardiomyocyte-related genes, potentially limiting insights into other cell types, a limitation that could be addressed by employing single-cell RNA sequencing.

Despite the limitations, the interest in LMSs technology is advancing rapidly, with ongoing developments focused on enhancing the applicability of the model and extending culturing conditions to support translational research. These advancements will refine disease models, improve reproducibility, and expand the range of cardiac conditions that LMSs can mimic. LMSs technology allows us to study a variety of parameters, including contractile function,^12^^,^^13^ calcium transients,^13^^,^^14^ and metabolism.^12^ Additionally, it enables the investigation of responses to mechanical preload and fibrotic reactions to different stimuli.^15^^,^^16^ In addition to this, our results demonstrate the potential of LMSs for studying angiogenic responses to gene therapy. Treatments with ZEB2 and TMSB4+PTMA increased the number of ECs, consistent with findings from *in vivo* studies, highlighting LMSs as a valuable tool for investigating vascular remodeling relevant to human heart disease, while the pro-angiogenic and cardioprotective potential of TMSB4 has been previously demonstrated in healthy pigs undergoing heart transplants,^17^ in a chronic porcine model of myocardial ischemia,^18^ and in pig-derived epicardial LMSs.^19^ Nevertheless, our study is the first report demonstrating the beneficial pro-angiogenic effects of ZEB2 and TMSB4+PTMA in pig-derived cardiac tissue, highlighting its capability to model complex pathological processes comparable to *in vivo* studies.

Porcine LMSs, in particular, provide a physiologically relevant model due to their close similarity to human cardiac tissue, including insights into cellular and molecular responses. However, species-specific differences between porcine and human systems underscore the need for careful interpretation and further validation using human-derived LMSs, which could provide an ideal translationally relevant model for preclinical research. This translation relevance was recently demonstrated by Abbas and colleagues.^20^ They showed that therapeutic inhibition of miR-21, which had previously been proven to prevent fibrotic responses in experimental animals, also provides functional benefits in LMSs from the human failing myocardium. An increasing number of similar studies are being conducted, paving the way for further research and establishing this technology as a valuable model for testing therapeutic applications. Still, additional studies are needed to fully validate the potential and further advance this model.

## Materials and methods

### LMSs preparation and functional testing

LMSs derived from the LV of pigs’ hearts were prepared similarly to previously described methods^13^ and were cultured in a transwell insert (Thermo Fisher Scientific, #140640).

### AAV preparation

AAV vectors were produced by the AAV Vector Unit at ICGEB Trieste. The viral concentration and incubation periods have been specified in the manuscript.

### RNA isolation, cDNA synthesis, and qPCR

Total RNA was isolated using ReliaPrep RNA Miniprep Systems (Promega, #Z6012), cDNA synthesis was performed using SuperScript II Reverse Transcriptase (Invitrogen, #18064014), and qPCR was performed on LightCycler 480 Instrument II (Roche, #05015243001) using LightCycler 480 SYBR Green I Master (Roche, #04707516001) according to the manufacturer’s protocols. Cq values were normalized to *Hprt1*. The primers for *Zeb2*, *GFP*, *Tmsb4*, *Ptma*, and *Hprt1* were designed as described previously,^6^ and sequences are available upon request.

### Immunofluorescence

4% PFA-fixed LMSs were processed, blocked with 4% BSA, and incubated with primary antibodies in 2% BSA. The following antibodies were used: anti-TNNT2 (Invitrogen, #MA5-12960, 1:200), anti-TNNT2 (Hytest, #4T21/2, 1:200), anti-GFP (AvesLabs, #GFP-1020, 1:200), anti-PECAM1 (Abcam, #AB28364, 1:50), and anti-KI67 (BD Pharma, #566003, 1:200). Corresponding Alexa Fluor secondary antibodies from Invitrogen (1:400) were used as described previously.^6^ Nuclei were counterstained with DAPI (Serva, #18860, 1:1,000). Images were captured using a Leica DM6000 and Leica TCS SP8 X white light laser confocal microscope. Quantification of immunofluorescence was conducted using ImageJ.

### Western blot

Tissue was lysated using radioimmunoprecipitation assay (RIPA) buffer supplemented with protease inhibitor (Roche, #11697498001, 1:50). Protein samples were denaturated before performing SDS-PAGE as previously described.^21^ The following antibodies were used: anti-ZEB2 (Novus Biological, #NBP1-77179, 1:1000), anti-CNX (Calbiochem, #208880, 1:2000) and corresponding horseradish peroxidase (HRP)-conjugated antibodies from BrightVision (1:2000). Proteins were detected using enhanced chemiluminescence (ECL) (Cytiva Amersham, #RPN2232) and visualized by ImageQuant LAS 4000 (GE Healthcare).

### Animal study

Mouse studies were conducted by protocols approved by the ethics committee of the Amsterdam UMC. Adult C57BL/6N male mice were housed in the standard condition with food and water *ad libitum*. AAV9 was delivered by intracardiac injections of a total dose of 0.5 × 10^11^ vg per mouse as we previously described.^6^

### RNA sequencing and KEGG pathway analysis

Total RNA was used for library preparation (KAPA Total RNA Hyperprep-RiboErase) and RNA sequencing (Illumina NovaSeq). KEGG pathway analysis was performed using DAVID.^22^

### Statistical analysis

Data are shown as mean ± standard error of the mean (SEM). Statistical analyses were performed using PRISM (GraphPad Software, v.9) using the Student’s t test and one-way ANOVA comparison test. Asterisks indicate statistical significance (∗*p* < 0.05, ∗∗*p* < 0.01, and ∗∗∗*p* < 0.001).

## Data availability

RNA sequencing data can be obtained from the authors upon request.

## Acknowledgments

This work is supported by the Dr. Dekker Senior Scientist Fellowship from the Dutch Heart Foundation (NHS2020T041 to M.M.G.), Horizon 2020 Eurostars (E114245 and E115484 to G.J.J.B.), the Dutch Research Council Open Technology Program 18485 (to G.J.J.B.), and the 10.13039/100018703European Innovation Council (EIC; PATHFINDER - Project 101115295 - NaV1.5-CARED and TRANSITION - Project 101099608 – TRACTION to G.J.J.B.).

## Author contributions

R.C. and M.M.G. designed the experiments. R.C., A.H., M.L.L., and A.R.B. performed the experiments. R.C. and M.M.G. analyzed the data. L.Z. and M.G. provided the AAV vectors. R.C., A.H., and M.M.G. wrote the manuscript. R.C., A.H., M.L.L., A.R.B, L.Z., G.J.J.B., M.G., and M.M.G. reviewed the manuscript.

## Declaration of interests

G.J.J.B. reports ownership interest in PacingCure BV. A.R.B. and G.J.J.B. are employees of PacingCure BV. M.M.G. is an associate editor of *Molecular Therapy*.
